# The Role of Vitamin D in Neuropathic Pain: Biological Mechanisms and Clinical Relevance

**DOI:** 10.3390/ijms27114671

**Published:** 2026-05-22

**Authors:** Mario García-Domínguez

**Affiliations:** Facultad de Educación, Universidad Alfonso X El Sabio (UAX), Avenida de la Universidad 1, Villanueva de la Cañada, 28691 Madrid, Spain; marigado@uax.es

**Keywords:** neuropathic pain, vitamin D, neuroinflammation, peripheral neuropathy, central sensitization, vitamin D receptor, neuroimmune modulation

## Abstract

Neuropathic pain remains a major clinical challenge due to its complex pathophysiology and limited treatment efficacy. Recent evidence suggests that vitamin D, beyond its classical role in bone and mineral metabolism, exerts neuroprotective and immunomodulatory effects that may influence pain perception. This review synthesizes current findings on the relationship between vitamin D status and neuropathic pain, highlighting potential mechanisms such as modulation of neuroinflammation, regulation of neuronal excitability, and influence on neurotransmitter pathways. Observational studies frequently report an association between vitamin D deficiency and increased pain severity, while interventional trials indicate that supplementation may alleviate neuropathic symptoms in specific populations. However, results remain heterogeneous, and mechanistic studies are still emerging. Understanding the interplay between vitamin D and neuropathic pain could open new avenues for adjunctive therapeutic strategies and personalized medicine approaches. Further high-quality clinical trials and mechanistic research are warranted to clarify causality and optimize clinical applications.

## 1. Introduction

Neuropathic pain is a chronic, debilitating condition resulting from injury to or disease of the somatosensory nervous system, involving both peripheral and central neural pathways [1]. Distinct from nociceptive pain, neuropathic pain is driven by dysfunctional neurobiological processes, like aberrant neuronal excitability, peripheral and central sensitization, neuroimmune dysregulation, and maladaptive synaptic plasticity [2,3,4]. Clinically, neuropathic pain manifests through heterogeneous symptom profiles like spontaneous pain, burning and shooting sensations, electric-shock-like episodes, allodynia, hyperalgesia, paresthesia, and sensory deficits [5]. These symptoms usually persist beyond tissue healing and disease resolution, reflecting sustained alterations in neural processing rather than ongoing peripheral injury [6]. Neuropathic pain is commonly associated with conditions such as diabetic neuropathy (DPN) [7], chemotherapy-induced peripheral neuropathy (CIPN) [8], postherpetic neuralgia [9], multiple sclerosis [10], and spinal cord injury [11], and it represents a major contributor to long-term disability, psychological distress, and reduced quality of life across diverse patient populations [12,13].

Existing pharmacological treatments (e.g., gabapentinoids, tricyclic antidepressants, serotonin-norepinephrine reuptake inhibitors, and opioids) modulate neuronal excitability and neurotransmission but often provide incomplete relief and are limited by side effects, tolerance, polypharmacy risks, and long-term safety concerns [14,15]. Interventional and neuromodulatory strategies, including spinal cord stimulation and nerve blocks, offer benefit in selected cases but remain invasive, costly, and not universally accessible [16,17]. Non-pharmacological approaches, such as cognitive-behavioral therapy, physical rehabilitation, and lifestyle interventions, facilitate multimodal management but rarely achieve sufficient symptom control when used in isolation [18,19,20]. Collectively, these limitations reflect a critical gap in current treatment paradigms, which primarily focus on symptom suppression rather than modification of underlying disease mechanisms.

In parallel with these clinical challenges, there has been increasing recognition of the role of neuroimmune interactions and neuroinflammation in the pathogenesis of neuropathic pain [21]. Peripheral and central insults trigger immune cell infiltration, microglial and astrocytic activation, and the release of pro-inflammatory cytokines and neurotoxic mediators [22,23]. These events drive long-lasting neuronal hyperexcitability, altered ion channel expression, compromised neurotransmitter release, and dysfunctional plasticity within pain-processing circuits [24,25,26,27]. Key molecular pathways (like NF-κB, MAPK signaling, and inflammasome activation) support the maintenance of chronic pain states [28]. As such, neuropathic pain is increasingly understood not merely as a disorder of neurons, but as a complex neuroimmune disease involving dynamic interactions between the nervous and immune systems.

Within this evolving mechanistic framework, vitamin D been recognized as a biologically relevant modulator of both neural and immune function. Beyond its classical endocrine role in Ca^2+^ and bone metabolism, vitamin D functions as a neuroactive steroid hormone, exerting genomic and non-genomic effects through the vitamin D receptor (VDR), which is expressed in neurons, glial cells, and several immune populations [29,30]. Vitamin D regulates transcriptional pathways involved in inflammatory signaling, oxidative stress control, mitochondrial function, and cellular survival [31,32]. Mechanistically, it has been shown to block pro-inflammatory cytokines including TNF-α, IL-1β, and IL-6, while promoting anti-inflammatory mediators like IL-10 [33]. It also modulates microglial activation states, reduces astrocytic reactivity, and influences neurotrophic factor expression, including NGF and BDNF, which are critical regulators of neuronal plasticity and regeneration [34,35,36].

At the level of neural excitability, vitamin D has been implicated in the regulation of Ca^2+^ and Na^+^ channel activity, glutamatergic and GABAergic neurotransmission, and synaptic signaling pathways that contribute to central sensitization [37]. Experimental studies suggest that vitamin D might decrease excitotoxicity, stabilize neuronal membranes, and modulate pain transmission within the dorsal horn and supraspinal pain-processing regions [38,39,40]. In several peripheral nerve injury models, vitamin D has shown potential neuroprotective effects, promoting axonal regeneration, Schwann cell function, and remyelination, while attenuating inflammatory damage to neural tissues [41,42,43,44]. These mechanistic actions directly intersect with core processes underlying neuropathic pain, including neuroinflammation, neuronal hyperexcitability, and maladaptive plasticity.

Clinically, the relevance of these mechanisms is supported by growing evidence linking vitamin D deficiency with chronic pain conditions, like neuropathic pain syndromes. Observational studies have reported associations between low serum 25-hydroxyvitamin D levels and increased pain severity, higher analgesic requirements, impaired functional outcomes, and poorer quality of life in patients with DPN, multiple sclerosis, and chronic neuropathic pain disorders [45,46,47]. Interventional studies, although heterogeneous in design and quality, show that vitamin D supplementation may improve pain scores, sensory symptoms, and functional status in selected patient populations [48,49,50]. While causality remains to be definitively established, these clinical findings align with mechanistic data and support the hypothesis that vitamin D may influence both the biological drivers and clinical expression of neuropathic pain.

This narrative review provides an updated and integrative synthesis of the biological pathways linking vitamin D to neuroimmune regulation and pain processing, as well as the experimental and clinical evidence supporting its role in neuropathic pain states. Unlike previous reviews, which have largely focused on isolated mechanistic aspects or limited clinical observations, this work integrates mechanistic, translational, and clinical data within a unified and structured framework. In addition, it evaluates the clinical relevance of vitamin D as an interesting therapeutic strategy in neuropathic pain. Importantly, this synthesis identifies critical research priorities, such as mechanistic investigations, the discovery and validation of reliable biomarkers, and the design of rigorous, well-powered, and methodologically robust clinical trials to establish causality and therapeutic efficacy.

## 2. General Characteristics of Vitamin D

### 2.1. Sources of Vitamin D

Vitamin D, a fat-soluble secosteroid, is obtained from both endogenous synthesis and exogenous sources. The primary endogenous source is cutaneous synthesis, where UVB radiation converts 7-dehydrocholesterol in the skin to previtamin D3, which subsequently undergoes thermally induced isomerization to vitamin D3 (known as cholecalciferol) [51]. Dietary sources, although generally less significant than endogenous production, include fatty fish including salmon, mackerel, and sardines, fish liver oils, egg yolks, and fortified foods like dairy-derived foods, cereals, and plant-based milk alternatives [52]. Ergocalciferol (vitamin D2), predominantly derived from fungal and yeast sources exposed to UV light, serves as an additional dietary form [53]. Environmental, geographical, and lifestyle factors (e.g., latitude, season, skin pigmentation, clothing, and sun exposure habits) influence the relative contribution of cutaneous versus dietary vitamin D, often leading to variability in population status [54,55].

### 2.2. Chemistry of Vitamin D

**Figure 1:**
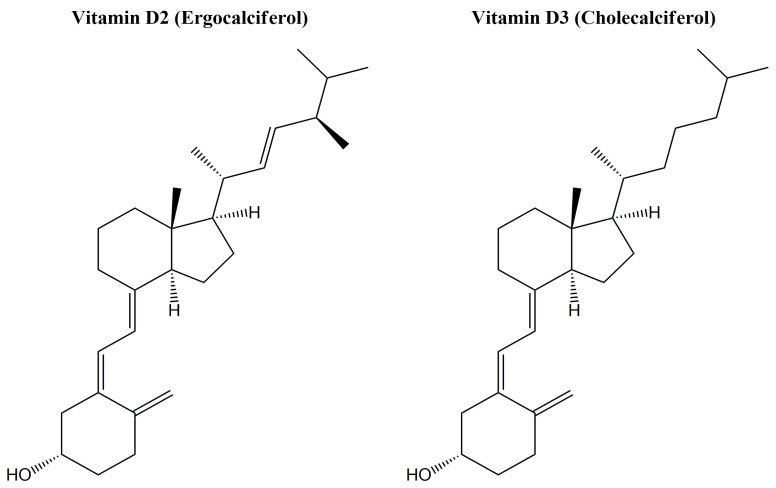
Chemical structures of vitamin D2 (ergocalciferol) and vitamin D3 (cholecalciferol).

Vitamin D comprises a chemically diverse group of fat-soluble secosteroids defined by a characteristic cleavage of the B-ring within the steroid nucleus, producing a secosteroid configuration that confers a huge flexibility and biological functionality [56]. The two principal vitamins relevant to human physiology, vitamin D3 and vitamin D2, differ structurally in the composition of their aliphatic side chains (Figure 1) [57]. These differences translate into distinct intramolecular architectures and energy landscapes. The C22-C23 double bond in ergocalciferol introduces π-electron density into the side chain, generating localized regions of electron delocalization that alter the electrostatic potential map of the molecule [58]. This unsaturation perturbs σ-π orbital interaction along the aliphatic side chain, leading to redistribution of electron density, altered bond polarization, and modified local electronic environments [59]. Consequently, vitamin D2 exhibits altered dipole vector orientation and anisotropic polarizability relative to vitamin D3, thereby influencing molecular conformation, metabolic stability, and binding affinity for vitamin D-binding protein (VDBP) and the vitamin D receptor (VDR) [60,61].

On the other hand, in vitamin D2, the presence of the C24 methyl substituent (absent in vitamin D3) promotes steric crowding and side-chain branching effects, leading to an increased van der Waals volume and enhanced molecular surface roughness within the terminal region of the aliphatic chain [62]. This additional steric bulk displaces molecular mass outward from the secosteroidal core, thus increasing the radius of gyration, expanding the solvent-accessible surface area, and reshaping the hydrophobic surface topology relative to vitamin D3 [63]. At the level of internal geometry, this branching perturbs bond angles and dihedral angle distributions along the C20-C27 segment, giving rise to distinct torsional strain profiles and alternative low-energy conformational minima within the potential energy surface that are not observed in vitamin D3 [64].

In addition, these substitutions influence intramolecular dispersion interactions and steric-electronic coupling between the side chain and the seco-B-ring core, subtly reshaping the overall three-dimensional molecular envelope [65]. From a computational perspective, these differences are reflected in distinct low-energy conformer populations, altered molecular flexibility indices, and variations in shape persistence parameters [66]. Vitamin D2 therefore occupies a different conformational ensemble than vitamin D3, characterized by reduced conformational entropy, increased steric anisotropy, and modified electronic density distribution [67].

From a physicochemical and pharmaceutical perspective, vitamin D is characterized by notable chemical instability [68]. Its secosteroid structure renders it susceptible to photodegradation, oxidative reactions, and acid-catalyzed isomerization, leading to the formation of biologically inactive or structurally altered derivatives [69]. These degradation pathways pose significant challenges for pharmaceutical formulation, dietary supplementation, and food fortification strategies [70,71]. As a result, stabilization approaches (like encapsulation technologies, antioxidant incorporation, oxygen-restricted packaging, and light-protective storage systems) are crucial to maintain compound integrity and biological efficacy in commercial and clinical applications [72,73].

Finally, the pronounced lipophilicity of vitamin D plays a crucial role in its absorption, distribution, and long-term bioavailability [74]. Intestinal uptake occurs via bile salt-dependent micellar solubilization and incorporation into chylomicrons, promoting lymphatic transport and distribution [75]. Following assimilation, vitamin D is sequestered in adipose tissue and skeletal muscle, forming a substantial extravascular reservoir that contributes to its prolonged half-life and buffering capacity against fluctuations in intake or endogenous synthesis [76]. However, this storage mechanism further aggravates pharmacokinetic modeling and contributes to interindividual variability in vitamin D status, particularly in the context of obesity, metabolic disorders, and malabsorptive conditions [77].

Both vitamin D3 and vitamin D2 function as inactive prohormones that require sequential enzymatic activation to exert biological effects [78]. Following cutaneous synthesis or intestinal absorption, vitamin D is primarily transported in the circulation bound to VDBP, which regulates its bioavailability and tissue distribution [79]. Hepatic 25-hydroxylation, mainly mediated by cytochrome P450 enzyme CYP2R1, yields 25-hydroxyvitamin D [25(OH)D] (calcidiol), the primary circulating metabolite and the most widely accepted biomarker of vitamin D nutritional status [80]. Following this, renal 1α-hydroxylation by CYP27B1 produces 1,25-dihydroxyvitamin D [1,25(OH)_2_D] (calcitriol), the hormonally active metabolite that binds to the VDR [81,82]. This endocrine activation pathway is regulated by parathyroid hormone (PTH), Ca^2+^ and PO_4_^3−^ concentrations, fibroblast growth factor 23 (FGF23), and feedback inhibition by 1,25(OH)_2_D itself, representing its incorporation into mineral and endocrine homeostasis [83].

### 2.3. Biological Functions and Mechanisms of Action of Vitamin D

Vitamin D exerts broad pleiotropic effects that extend far beyond its classical role in Ca^2+^ and PO_4_^3−^ homeostasis. Its biologically active metabolite (calcitriol) mediates most of its effects via the VDR, a member of the nuclear receptor superfamily [84]. In the intestine, VDR activation promotes the enhancement of dietary Ca^2+^ absorption [85]; in bone, vitamin D modulates osteoblast and osteoclast activity, supporting bone mineralization and remodeling [86]; in the kidneys, VDR regulates Ca^2+^ reabsorption and PO_4_^3−^ metabolism, contributing to systemic mineral balance [87]; in the immune system, vitamin D promotes immunomodulatory effects, enhancing innate immunity and regulating adaptive immune responses by modulating T cell differentiation and cytokine production [88]; in the cardiovascular system, vitamin D influences vascular tone, inhibits inflammation, and may protect against endothelial dysfunction [89]; in adipose tissue, vitamin D regulates energy metabolism and adipokine regulation [90]; in the CNS, vitamin D has neuroprotective roles, affecting neuronal differentiation, neurotransmission, and the modulation of neurotrophic factors [91].

**Figure 2:**
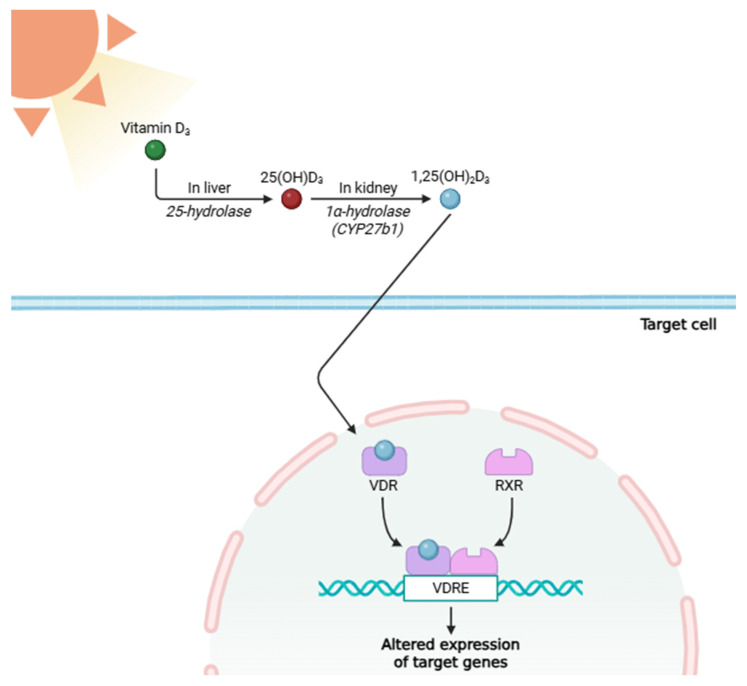
Cholecalciferol is metabolically activated through sequential hydroxylation in the liver to 25(OH)D_3_ (calcidiol) and in the kidney to 1,25(OH)_2_D_3_ (calcitriol). The active form binds to the VDR, forming a heterodimer with the RXR, which interacts with VDREs in DNA to promote gene expression. Abbreviations: 25(OH)D_3_ (25-hydroxyvitamin D_3_, calcidiol); 1,25(OH)_2_D_3_ (1,25-dihydroxyvitamin D_3_, calcitriol); CYP27B1 (cytochrome P450 family 27 subfamily B member 1); VDR (vitamin D receptor); RXR (retinoid X receptor); VDRE (vitamin D response element).

Upon binding of calcitriol (Figure 2), the VDR undergoes a conformational change that exposes its DNA-binding and ligand-binding domains, allowing heterodimerization with the retinoid X receptor (RXR) [92]. The VDR-RXR complex translocates into the nucleus, where it specifically binds vitamin D response elements (VDREs) within promoter and enhancer regions of target genes to regulate transcription [93]. The VDR-RXR heterodimer translocates to the nucleus, where it selectively interacts with VDREs within promoter and enhancer regions of several target genes to modulate transcriptional activity [94,95]. Ligand-bound VDR recruits some transcriptional coactivators, such as steroid receptor coactivators (SRC-1, SRC-2, and SRC-3), histone acetyltransferases (p300/CBP), and several components of the mediator complex, which collectively facilitate chromatin remodeling, nucleosome repositioning, and RNA polymerase II recruitment [96,97,98,99]. In the absence of ligands, VDR preferentially associates with corepressors such as nuclear receptor corepressor (NCoR) and silencing mediator for retinoid and thyroid receptors (SMRT), which mobilize several histone deacetylases (HDACs), resulting in transcriptional repression [100]. These interactions allow VDR to finely modulate gene expression depending on ligand availability, cellular context, and tissue type.

Through these canonical genomic pathways, VDR regulates many physiological processes. In the intestine, VDR activation induces expression of Ca^2+^ channels (TRPV6), intracellular Ca^2+^-binding proteins (calbindin-D9k and calbindin-D28k), and basolateral calcium pumps (PMCA1b), enabling dietary Ca^2+^ absorption [75,85]. In the kidney, VDR controls Ca^2+^ reabsorption via TRPV5 and calbindin-D28k and phosphate handling through regulation of NaPi-IIa and NaPi-IIc transporters [101]. Within bone, VDR modulates osteoblast differentiation and matrix protein synthesis, including osteocalcin and osteopontin, while indirectly regulating osteoclast activity through transcriptional control of RANKL and osteoprotegerin (OPG), thus maintaining skeletal integrity and mineralization [102]. Beyond mineral metabolism, VDR signaling has profound immunomodulatory effects. In innate immune cells, like monocytes and dendritic cells, VDR induces expression of antimicrobial peptides, including cathelicidin and defensins, enhances pathogen recognition, and coordinates Toll-like receptor (TLR) signaling [103,104]. In adaptive immunity, VDR suppresses the differentiation of pro-inflammatory Th1 and Th17 cells while promoting regulatory T cell development, shaping cytokine profiles toward an anti-inflammatory state with increased IL-10 and decreased IFN-γ, IL-2, and IL-17 production [105,106].

In addition to canonical genomic pathways, VDR mediates rapid, non-genomic signaling through membrane-associated or caveolae-localized receptors. These non-genomic actions activate intracellular kinase cascades, including PI3K/Akt, MAPK/ERK, PKC, and p38 MAPK, and rapidly modulate intracellular Ca^2+^ flux, mitochondrial function, and reactive oxygen species (ROS) production [107,108].

VDR also regulates cellular proliferation, differentiation, and apoptosis through transcriptional modulation of cell cycle and many apoptotic genes [109]. Ligand-bound VDR induces cyclin-dependent kinase inhibitors (p21 and p27), leading to G1/S cell cycle arrest, while controlling apoptosis via regulation of BCL2 family proteins and caspase activation [110]. These mechanisms contribute to tissue regeneration, epithelial barrier maintenance, and tumor suppression [111].

Finally, epigenetic regulation is another key aspect of VDR signaling. Ligand-bound VDR recruits histone acetyltransferases and methyltransferases, promoting activating histone modifications including H3K9ac, H3K27ac, and H3K4me3, which enhance chromatin accessibility [112]. Concurrently, VDR can displace corepressors and histone deacetylases, or interact with repressive methyltransferases to inhibit transcription [113]. VDR also interacts with ncRNAs, such as miRNAs and lncRNAs, introducing another post-transcriptional layer of regulation [114].

### 2.4. Epidemiology of Vitamin D Status

Vitamin D deficiency constitutes a global public health concern, with prevalence patterns shaped by complex interactions among geographic latitude, solar UVB radiation exposure, dietary intake, cultural practices, and socio-demographic determinants [115]. Epidemiological evidence consistently demonstrates disproportionately higher rates of deficiency in populations residing at higher latitudes, among individuals with increased skin melanin content, and within urbanized, institutionalized, or socioeconomically disadvantaged cohorts [116,117]. Serum calcidiol concentrations below 20 ng/mL are widely recognized as indicative of deficiency, whereas levels ranging from 21 to 29 ng/mL are classified as insufficiency according to international clinical guidelines [118].

Vitamin D deficiency represents a significant public health concern with wide-ranging clinical implications that extend beyond classical skeletal pathology. At the osteometabolic level, long-lasting hypovitaminosis D disrupts Ca^2+^-PO_4_^3−^ homeostasis, impairing bone mineralization and leading to well-established disorders like nutritional rickets in pediatric populations, osteomalacia in adults, and secondary hyperparathyroidism [119]. Moreover, via its regulatory role in bone remodeling and mineral density maintenance, vitamin D deficiency contributes to the pathophysiology of osteoporosis, thereby increasing fracture risk and skeletal fragility, particularly among aging populations [120,121].

Beyond musculoskeletal health, growing evidence indicates that suboptimal vitamin D status is linked to multisystem dysfunction and increased susceptibility to a broad spectrum of chronic diseases. Epidemiological and mechanistic studies link vitamin D deficiency to immune dysregulation, chronic low-grade inflammation, and altered endocrine signaling, which might predispose individuals to autoimmune diseases (such as multiple sclerosis and type 1 diabetes mellitus), increased infectious morbidity, metabolic dysfunctions including insulin resistance and metabolic syndrome, adverse cardiovascular outcomes, and certain malignancies [122,123,124,125].

Importantly, clinical manifestations of vitamin D deficiency are highly heterogeneous, reflecting the complexity of vitamin D biology and its interaction with multiple modifying factors, including genetic variability, comorbid conditions, nutritional status, lifestyle behaviors, sun exposure, and broader socio-environmental determinants [126]. Consequently, hypovitaminosis D should not be conceptualized merely as an isolated nutritional deficiency, but rather as a systemic biological modifier that may contribute to multisystem disease vulnerability and the progression of chronic pathological processes [127]. This broader perspective reinforces the relevance of vitamin D as a critical determinant in preventive medicine, population health strategies, and integrative models of chronic disease prevention [128].

Extensive epidemiological surveillance networks and rigorously characterized population-based cohorts, including the National Health and Nutrition Examination Survey (NHANES), represent invaluable resources for the assessment of vitamin D status across diverse populations [129]. These datasets provide both longitudinal and cross-sectional insights, enabling researchers to characterize patterns of calcidiol concentrations across different demographic strata, such as age, sex, ethnicity, and socioeconomic status, as well as geographic regions and seasonal fluctuations [130]. The depth and breadth of these data allow for the identification of high-risk subgroups with insufficient or deficient vitamin D levels, facilitating a more nuanced understanding of the determinants of vitamin D status, including dietary intake, sunlight exposure, lifestyle factors, comorbid conditions, and genetic polymorphisms influencing vitamin D metabolism [131,132].

### 2.5. Supplementation Strategies and Correction of Deficiencies

Vitamin D supplementation constitutes a fundamental intervention for the correction of deficiency and the maintenance of optimal serum calcidiol and calcitriol concentrations.

The dosing strategy is typically individualized based on factors such as chronological age, baseline serum calcidiol levels, presence of comorbidities (such as chronic kidney disease and malabsorption syndromes), body mass index, and recognized risk factors for hypovitaminosis D, such as limited sun exposure, darker skin pigmentation, and advanced age [118,128,133].

Oral supplementation with vitamin D3 is generally favored over vitamin D2 due to its superior bioefficacy, more predictable pharmacokinetics, and longer duration of action [134]. Protocols for supplementation range from daily low-dose regimens (typically 400–2000 IU per day) to intermittent high-dose strategies, like 50,000 IU administered weekly or monthly, depending on clinical context [133,135]. Independent of the supplementation protocol, serum calcidiol monitoring is essential to optimize efficacy and prevent toxicity, which may present as hypercalcemia, hypercalciuria, nephrolithiasis, or other renal complications [136].

At the population level, fortification of staple foods (e.g., dairy products, cereals, and edible oils) has demonstrated efficacy in reducing the prevalence of deficiency and supporting public health outcomes [137,138]. Personalized supplementation strategies must also consider variables that influence absorption and metabolism, including gastrointestinal disorders, adherence patterns, concomitant medications, and pharmacogenomic polymorphisms affecting vitamin D-related enzymes or the VDR [128,133].

Emerging research is exploring innovative approaches to enhance bioavailability and therapeutic outcomes, such as liposomal or nanoparticle-based formulations, sublingual preparations, and UVB-emitting devices designed to stimulate cutaneous synthesis [139,140,141]. These modalities aim to improve pharmacokinetic profiles, enhance tissue delivery, and minimize the risk of side effects, thus offering a more targeted and efficient approach to both prophylaxis and therapy of vitamin D deficiency.

## 3. Neurobiological Roles of Vitamin D

### 3.1. Vitamin D Metabolism in the Nervous System

Vitamin D biotransformation in the nervous system constitutes a highly specialized, autonomous neuroendocrine-like network, defined by strict cell-type specificity and subcellular compartmentalization, and tightly controlled enzymatic and signaling pathways.

Beyond passive diffusion, the uptake of circulating calcidiol into the CNS occurs through a receptor-mediated mechanism [142,143]. Although an exact mechanism has not yet been elucidated, transport across the blood–brain barrier (BBB) and blood-CSF barrier is probably facilitated by DBP-25(OH)D complexes through LRP2 (megalin)/cubilin-dependent endocytosis, expressed in brain microvascular endothelial cells, choroid plexus epithelial cells, and ependymal cells [144,145,146]. This process is regulated by endosomal acidification and clathrin-dependent vesicular trafficking, facilitating the intracellular release of calcidiol into the cytosol for subsequent enzymatic activation [145].

Neural cells possess a fully functional intracrine vitamin D metabolic machinery, enabling autonomous synthesis of the active hormone independent of renal endocrine regulation. Neurons and glial cells express CYP27B1, a mitochondrial cytochrome P450 enzyme that catalyzes the conversion of calcidiol to calcitriol [147,148]. CYP27B1 activity in neural cells is probably (the exact mechanisms in neural cells remain unknown) regulated by intracellular Ca^2+^ signaling [149]. Several pro-inflammatory cytokines (including IL-1β, TNF-α, IFN-γ, and IL-6) upregulate CYP27B1 expression in glial cells, connecting immune activation to enhanced local vitamin D bioactivation [150,151].

Finally, catabolic regulation is mediated by CYP24A1, an ER-associated enzyme that launches multistep hydroxylation of calcidiol and calcitriol into inactive metabolites, culminating in calcitroic acid formation [152]. CYP24A1 expression is upregulated by VDR-RXR signaling, establishing a canonical intracellular negative feedback loop that restricts the duration of vitamin D signaling, prevents ligand overaccumulation, and sustains transcriptional homeostasis [153].

### 3.2. VDR Expression in Neural Tissues

The VDR is widely expressed throughout the CNS and PNS, displaying a regionally enriched and cell-type-specific distribution. In the cerebral cortex, VDR expression is predominantly observed in pyramidal projection neurons, particularly within layers III and V, suggesting a prominent localization in cortical circuits [154,155]. Immunohistochemical and in situ hybridization studies have consistently identified VDR in neuronal somata and nuclei across frontal, parietal, and temporal cortical regions, demonstrating a broad cortical presence rather than confinement to a single functional domain [155,156].

Subcortically, VDR is detectable in dopaminergic neurons of the substantia nigra pars compacta and has also been observed in the ventral tegmental area [157]. In the cerebellum, VDR is expressed in Purkinje cells of the Purkinje layer and, to a lesser extent, in cells of the granular layer [154]. Within the hippocampus, VDR is markedly expressed in CA1, CA2, and CA3 pyramidal neurons as well as in dentate gyrus [158]. Moreover, expression has been reported in the subiculum and entorhinal cortex, further extending its distribution across limbic-associated regions [159].

Beyond neuronal populations, VDR expression extends to several glial cell types. Oligodendrocyte precursor cells (OPCs) and mature oligodendrocytes express VDR within white matter tracts and cortical regions, indicating its presence across the oligodendroglial lineage [160,161]. Astrocytes throughout gray and white matter also show VDR immunoreactivity, such as protoplasmic astrocytes in cortical areas and fibrous astrocytes in subcortical white matter [162]. On the other hand, VDR expression has been documented in microglial cells under both basal and activated conditions, supporting its presence within the neuroimmune component of the CNS [163].

Finally, in the PNS, VDR expression has been detected in dorsal root ganglion (DRG) neurons and in Schwann cells, indicating that its localization extends beyond the brain and spinal cord [164,165].

### 3.3. Neurodevelopmental Functions

Vitamin D exerts tightly coordinated control over neural progenitor dynamics during embryogenesis via VDR-dependent transcriptional programs working within ventricular and subventricular germinal zones. By orchestrating the expression of key cell-cycle regulators including cyclin D1, p21, p27, and p53, vitamin D controls progenitor cell proliferation and modulates the balance between symmetric self-renewal and asymmetric neurogenic division [166,167]. Mechanistically, upon ligand engagement, VDR associates with VDREs in the promoter regions of key cell-cycle regulatory genes, while concurrently governing regulatory control over the Wnt/β-catenin signaling pathway [168]. Suppression of nuclear β-catenin accumulation and reduced TCF/LEF transcriptional activity shift progenitor cells toward controlled cell-cycle exit and lineage commitment, thus preventing excessive proliferation and supporting orderly cortical expansion [169].

During neuronal differentiation, vitamin D strengthens the transcription of key neurotrophic factors, including NGF, BDNF, GDNF, and NT-3, creating a permissive microenvironment for neuronal survival and maturation [170,171]. Increased expression of Trk receptors further sensitizes differentiating neurons to trophic support, facilitating activation of intracellular cascades that upregulate cytoskeletal stabilization, metabolic competence, and resistance to apoptotic signaling [172]. Vitamin D also promotes axonal growth and neuronal migration by regulating genes implicated in cytoskeletal organization [173]. Upregulation of microtubule-associated proteins such as MAP2 and tau stabilizes axonal and dendritic processes, whereas modulation of actin-binding proteins including cofilin and profilin optimizes actin filament turnover [171]. Concurrent regulation of Rho family GTPases (RhoA, Rac1, and Cdc42) facilitates spatial control over growth cone dynamics, influencing motility, polarity establishment, and directional responsiveness to extracellular guidance cues [174].

### 3.4. Neurotransmission and Synaptic Plasticity

Synaptogenesis is influenced by vitamin D through its regulation of gene transcription underlying synaptic adhesion and connectivity. Vitamin D_3_, when binds to the VDR, forms a VDR-RXR complex that modulate the expression of key synaptic adhesion molecules, including neuroligins (NLGNs), neurexins (NRXNs), classical cadherins, and protocadherins, which are essential for synaptic specificity and structural stabilization [175,176,177].

At the molecular level, vitamin D-mediated upregulation of neurexins in presynaptic terminals and neuroligins in postsynaptic densities facilitates trans-synaptic signaling that ensures proper excitatory and inhibitory synaptic contacts [178]. Cadherins and protocadherins provide Ca^2+^-dependent adhesion cues that stabilize nascent synaptic contacts and support dendritic spine morphogenesis [179,180]. By fine-tuning these adhesion systems, vitamin D promotes activity-dependent synaptic refinement, enhancing the precision of excitatory glutamatergic and inhibitory GABAergic circuits [181,182].

### 3.5. Neuroprotection and Neuroimmune Modulation

Vitamin D, particularly in its biologically active form 1,25-dihydroxyvitamin D_3_ (calcitriol), exerts neuroprotective and immunomodulatory effects within the CNS. Vitamin D promotes neuronal survival through the transcriptional upregulation of anti-apoptotic genes such as BCL2 and the concomitant repression of pro-apoptotic mediators including BAX and caspase-3 [183,184]. Furthermore, vitamin D modulates intracellular Ca^2+^ homeostasis by regulating the expression of voltage-gated calcium channels (VGCCs) and calcium-binding proteins such as calbindin and parvalbumin, reducing excitotoxic vulnerability mediated by excessive glutamatergic stimulation and NMDA receptor overactivation [185,186,187]. Through these molecular mechanisms, vitamin D attenuates Ca^2+^-dependent mitochondrial damage and prevents cytochrome c release and apoptosome formation [188].

Vitamin D also produces potent antioxidant effects by enhancing the transcription of genes encoding key antioxidant enzymes, including glutathione peroxidase (GPx), superoxide dismutase (SOD), and catalase [189,190]. The stimulation of the Nrf2 (nuclear factor erythroid 2-related factor 2) pathway further amplifies the cellular antioxidant response, enhancing glutathione synthesis and detoxification of ROS [191]. By counteracting oxidative stress, vitamin D preserves mitochondrial membrane potential, maintains ATP production, and reduces lipid peroxidation and DNA damage, all of which are central contributors to neurodegenerative cascades [192].

From an immunological perspective, vitamin D acts as an essential regulator of neuroimmune homeostasis. In microglial cells, activation of the VDR promotes a phenotypic shift from the classically activated M1 state, which is characterized by a pro-inflammatory profile, toward the alternatively activated M2 state, linked to anti-inflammatory signaling, resolution of inflammation, and tissue repair [193]. The M1 phenotype is typically induced in response to pathogens, cellular damage, or pro-inflammatory stimuli and is characterized by the production of pro-inflammatory mediators (including IL-1β, IL-6, TNF-α, and IFN-γ), alongside suppression of inducible nitric oxide synthase (iNOS) and cyclooxygenase-2 (COX-2) [194]. Continuous activation of the M1 state contributes to chronic neuroinflammation, oxidative stress, neuronal dysfunction, and progressive tissue injury. In contrast, the M2 phenotype is associated with anti-inflammatory functions, resolution of inflammatory processes, and promotion of tissue repair. M2 microglia contribute to the restoration of homeostasis through the secretion of anti-inflammatory mediators such as IL-10 and TGF-β, as well as through an increased phagocytic capability, enabling more efficient clearance of cellular debris, apoptotic cells, and toxic protein aggregates. Moreover, M2 polarization promotes extracellular matrix remodeling, angiogenesis, and neuronal survival, thus promoting tissue regeneration and recovery following injury [195]. From a mechanistic perspective, vitamin D interferes with NF-κB signaling by promoting the stabilization of IκBα and inhibiting nuclear translocation of the p65 subunit, thus reducing transcription of inflammatory mediators [196]. Moreover, vitamin D reduces activation of the NLRP3 inflammasome, decreasing caspase-1 activation and the maturation of IL-1β and IL-18, which are key drivers of chronic neuroinflammation [197].

In astrocytes, vitamin D reduces reactive astrogliosis and modulates the secretion of chemokines and cytokines that govern leukocyte recruitment [198]. Vitamin D downregulates expression of major histocompatibility complex class II (MHC-II) and costimulatory molecules, thus limiting antigen-presenting capacity within the CNS [199]. Within adaptive immunity, vitamin D restrains Th1 and Th17 differentiation while facilitating regulatory T cell (Treg) expansion via modulation of IL-12, IL-23, and TGF-β signaling pathways [200,201]. This transition to an anti-inflammatory immune milieu is primarily relevant in autoimmune neuroinflammatory disorders, where aberrant Th17 responses contribute to demyelination and axonal injury [202].

Finally, vitamin D further contributes to neuroprotection by maintaining BBB integrity. It enhances expression of tight junction proteins such as claudin-5, occludin, and zonula occludens-1 (ZO-1), thereby decreasing endothelial permeability and limiting infiltration of peripheral immune cells [203,204]. Concurrently, vitamin D downregulates matrix metalloproteinases (e.g., MMP-9), which are involved in extracellular matrix degradation and BBB disruption during inflammatory states [205]. Preservation of BBB structure mitigates secondary neuroinflammatory amplification and protects neuronal networks from circulating toxins and immune-mediated damage [206].

## 4. Potential Mechanisms Linking Vitamin D to Neuropathic Pain

Neuropathic pain emerges from maladaptive alterations within the somatosensory system, whereby neural circuits that ordinarily transmit protective nociceptive signals undergo pathological reorganization, resulting in sustained neuronal hyperexcitability and chronic, self-amplifying neuroinflammation [1,2,3,4]. In this pathological context, cellular energy requirements increases substantially while homeostatic regulatory systems deteriorate, culminating in overlapping cascades of oxidative stress, immune activation, synaptic restructuring, and transcriptional plasticity [207]. Vitamin D occupies a central role in this transition as a pluripotent regulator, capable of integrating endocrine, immune, and neuronal systems into a unified modulatory axis [208].

Following peripheral nerve injury, axons and their myelin sheaths experience structural breakdown in a process known as Wallerian degeneration [209]. The immediate biochemical aftermath consists of Ca^2+^ influx, mitochondrial rupture, and cytoskeletal collapse within the distal axon [210]. This event releases mtDNA, ATP, and oxidized phospholipids, which act as danger-associated molecular patterns (DAMPs) to activate macrophages, Schwann cells, and microglia [211]. Under physiological conditions, this response is transient and resolves as debris is cleared and axonal repair progresses, with vitamin D playing a central role in maintaining this self-limiting nature. Through activation of the VDR, vitamin D upregulates the expression of antioxidative and detoxifying enzymes that limit ROS propagation, such as SOD, catalase, and glutathione peroxidase [189,190]. Vitamin D also promotes metabolic polarization of infiltrating macrophages from the glycolytic, ROS-producing M1 phenotype toward the oxidative, reparative M2 state, characterized by arginase-1 activity and the release of IL-10, VEGF, among others [212]. In contrast, vitamin D deficiency allows the inflammatory response to persist, sustaining pro-oxidant cytokine environments, impairing Schwann cell-mediated remyelination, and enabling unresolved oxidative injury to convert regenerating tissue into a source of continuous nociceptive signaling [213]. By preserving mitochondrial bioenergetic function and immune equilibrium during this interval, vitamin D indirectly limits the formation of a molecular landscape that promotes neuropathic sensitization [214].

Progressive alterations during axonal degeneration extend into the phase of peripheral sensitization, during which primary afferent neurons exhibit aberrant excitability. Injured nociceptors exhibit enhanced expression of voltage-gated sodium channels (VGSCs; Nav1.7-Nav1.9), transient receptor potential channels (TRPs; TRPV1 and TRPA1), and reduced expression of K^+^ channels that commonly stabilize the resting membrane potential [215]. These alterations are mediated by transcriptional and post-translational modifications induced by inflammatory factors including prostaglandins, bradykinin, and TNF-α [216]. Vitamin D mitigates these processes through both genomic and rapid non-genomic mechanisms, with the calcitriol-VDR complex binding to VDRE sequences within channel gene promoters to block SCN10A and SCN11A transcription while upregulating KCNQ2 and KCNB1, thereby elevating the depolarization threshold and limiting repetitive firing [143,217]. In parallel, membrane-associated VDR and PDIA3 activate PI3K/Akt and PKC signaling cascades, transiently reducing the open probability of L-type Ca^2+^ channels and preventing PKC-mediated phosphorylation of TRPV1, resulting in heightened nociceptor sensitivity to heat and H^+^ [218,219]. Within the biochemical milieu of peripheral sensitization, characterized by increased intracellular Ca^2+^, ATP, and NO that perpetuate self-reinforcing signaling loops, vitamin D functions a dynamic regulatory modulator, dampening excitatory channel activity while driving the production of Ca^2+^-binding proteins, mainly calbindin-D28k and parvalbumin [40,44,220]. Collectively, these molecular pathways increase nociceptive thresholds, attenuate ectopic firing, and limit aberrant action potential initiation in injured afferent neurons.

Sustained peripheral sensitization drives the amplification and maintenance of synaptic hyperexcitability within central nociceptive networks, a pathological process known as central sensitization [221]. This phenomenon encompasses multiple convergent mechanisms, including phosphorylation of NMDA receptor subunits [222], enhanced trafficking of AMPA receptors to the postsynaptic membrane [223], and disruption of inhibitory neurotransmission, with microglial activation in the dorsal horn serving as a fundamental intermediary [224]. Activated by ATP acting on P2X4 receptors and by neuronal CX3CL1, microglia release pro-inflammatory mediators (like TNF-α and IL-1β), and BDNF, which downregulates neuronal expression of the K^+^-Cl^−^ cotransporter KCC2, thereby inducing depolarizing shifts in GABAergic signaling and functionally converting inhibitory synapses into excitatory ones [225,226]. Vitamin D selectively counteracts this pathogenic triad by suppressing microglial activation markers (Iba1 and CD68) and restricting P2X4 receptor expression, resulting in reduced cytokine and BDNF release, while concurrently preserving KCC2 transcription to maintain the hyperpolarizing properties of GABAergic currents and prevent the loss of inhibitory control [227,228]. In addition, calcitriol upregulates astrocytic glutamate transporters EAAT1/2, enabling clearance of excess synaptic glutamate, and restores redox balance through activation of Nrf2, thus protecting glutamate-cysteine ligase (an essential enzyme for glutathione synthesis) from oxidative inactivation [191,229]. These coordinated molecular effects block the long-term potentiation-like cellular processes that drive central pain amplification.

**Figure 3:**
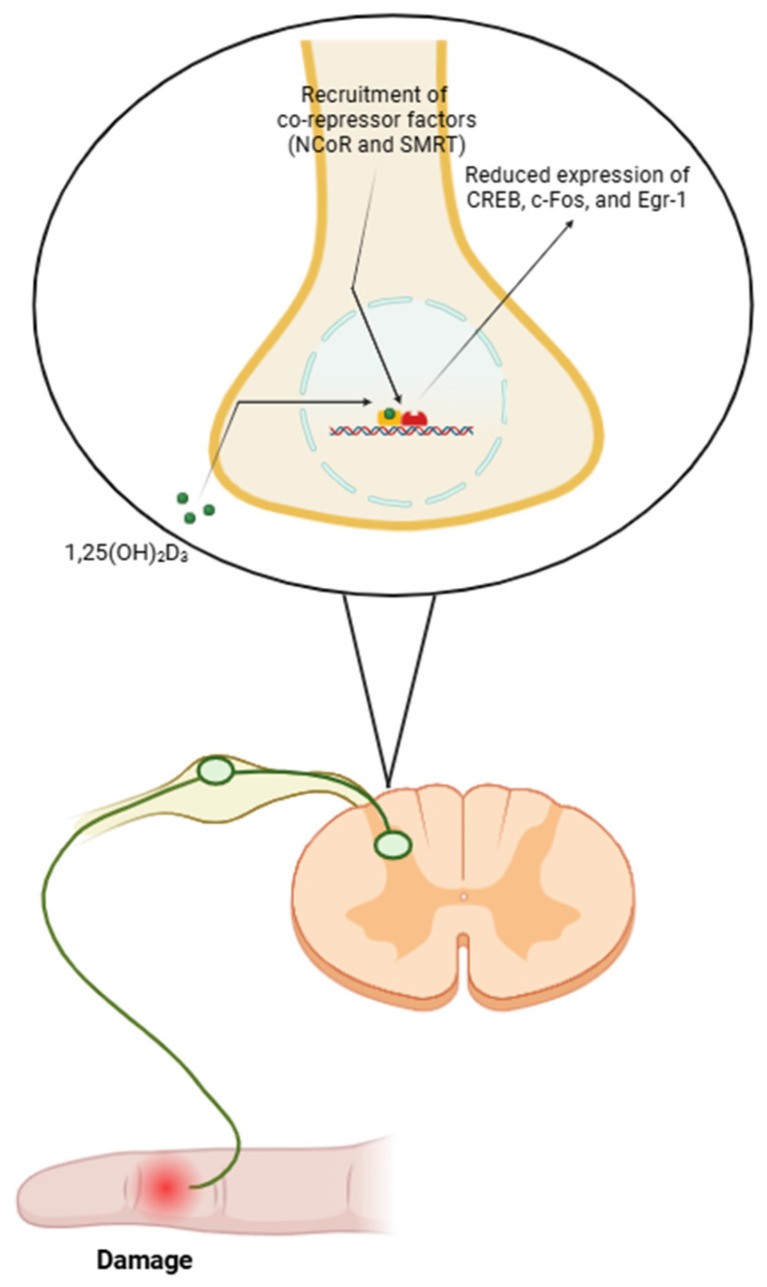
The active vitamin D metabolite (calcitriol) modulates neuronal responses to peripheral injury by promoting the recruitment of co-repressor complexes (NCoR and SMRT) at the nuclear level. This interaction suppresses the expression of key transcription factors, including CREB, c-Fos, and Egr-1, leading to reduced gene activation associated with neuronal excitation and inflammation, and suggesting a neuroprotective and anti-nociceptive role. Abbreviations: 1,25(OH)_2_D_3_ (1,25-dihydroxyvitamin D_3_, calcitriol); NCoR (nuclear receptor co-repressor); SMRT (silencing mediator of retinoid and thyroid receptors); CREB (cAMP response element-binding protein); c-Fos (FBJ murine osteosarcoma viral oncogene homolog); Egr-1 (early growth response protein 1).

An often-overlooked dimension of central sensitization is the reorganization of gene expression within dorsal-horn neurons under persistent stimulation. Transcription factors such as CREB, c-Fos, and Egr-1 become chronically activated, ensuring continued production of excitatory receptors and signaling molecules [230,231]. Experimental findings from cultured cells suggest that vitamin D may modulate this transcriptional hyperactivity via recruitment of nuclear co-repressors NCoR and SMRT, which deacetylate histones at promoters of these genes, and by induction of sirtuin-1, driving chromatin condensation and metabolic resilience (Figure 3) [232]. In nociceptors, the calcitriol-VDR complex has also been proposed to interact with PGC-1α, coupling transcriptional regulation to mitochondrial energy status; however, the relevance of this mechanism in vivo in neuropathic pain models remains incompletely characterized [233]. Through these mechanisms, vitamin D may contribute to the repression of maladaptive transcriptional programs associated with persistent pain memory.

As central sensitization progresses, glial plasticity becomes the sustaining infrastructure of neuropathic pain. Astrocytes and microglial cells form a self-reinforcing neuroimmune network that sustains excitatory drive via cytokines, ATP, and NO [234,235]. Vitamin D has been reported to modulate glial cross-communication, including suppression of connexin 43 expression and restriction of gap junction coupling that enables Ca^2+^ wave propagation across astrocytic domains [151,229]. Simultaneously, vitamin D may enhance astrocytic glutamine synthetase activity, potentially improving glutamate recycling into glutamine and reducing extracellular excitatory load [236]. Microglia under the influence of vitamin D have been shown in experimental studies to display constrained expression of the NADPH oxidase NOX2 complex alongside increased mitochondrial oxidative enzyme activity, which may mitigate the oxidative tone sustaining their reactive state [237]. Furthermore, vitamin D has been associated with metabolic reprogramming of microglial phenotypes, including promotion of mitochondrial biogenesis and β-oxidation, changes commonly linked to anti-inflammatory M2-like functional profiles [238]. This shift from glycolytic to oxidative metabolism drives a functional transition from cytokine-producing effector cells to neurotrophic-supportive guardians, showing that vitamin D reshapes not only glial gene expression but also their metabolic behavior and intercellular communication.

In the spinal cord, excessive glial activity can propagate to supraspinal regions, generating aberrant circuit reorganization within thalamic and cortical regions (a phenomenon translated clinically into hyperalgesia and allodynia) [239,240]. Vitamin D allows synaptic pruning and remyelination that oppose this aberrant propagation, inducing the expression of synaptic stabilizers such as synapsin I and PSD-95 to promote balanced synaptic maintenance, while VDR-mediated upregulation of some myelin structural genes in oligodendrocytes accelerates the normalization of conduction velocity, and together these central restorative mechanisms complete the final loop through which vitamin D prevents the consolidation of chronic pain at the level of cortical representations [241,242].

The theoretical implications are substantial. Vitamin D signaling can be conceptualized as a higher-order regulator of the nociceptive network, functioning as a biochemical constraint that reduces entropy across interconnected systems involved in nerve injury, immune activation, and neural plasticity. Deficiency in vitamin D disrupts this regulatory constraint, permitting stochastic fluctuations and maladaptive feedback loops to predominate in nociceptive signal processing. Consequently, the normally protective nociceptive system may transition into a pathological, self-sustaining excitatory state.

## 5. Vitamin D and Neuropathic Pain: Preclinical and Clinical Evidence

### 5.1. Preclinical Evidence

Preclinical research spanning over two decades has unequivocally established vitamin D, primarily administered as vitamin D3, as a potent modulator of neuropathic pain behaviors, biochemical cascades, and structural neuropathology in many rodent models, ranging from traumatic peripheral nerve injuries to toxic, metabolic, and inflammatory neuropathies (Table 1). Such prolonged exposure is essential in facilitating hepatic 25-hydroxylation and renal activation to 1,25-dihydroxyvitamin D3, which joins VDRs in some cell types.

The research trajectory was initiated through early experimental work employing the chronic constriction injury (CCI) model of the sciatic nerve, originally developed by Bennett and Xie in 1988 [249]. This paradigm reproduces core pathophysiological processes underlying human compressive neuropathies, including aberrant ectopic firing, Wallerian degeneration, and central sensitization, as observed in clinical entities such as lumbar radiculopathy and trigeminal neuralgia. Male Sprague Dawley rats undergoing unilateral CCI were treated with intraperitoneal vitamin D3 at 1000 IU/kg per day starting on postoperative day 1 and maintained for 21 consecutive days, a dosing regimen that significantly attenuated both thermal hyperalgesia and cold allodynia [243]. Further developing these findings, subsequent investigation utilizing the CCI model in Wistar rats delivered oral vitamin D3 (500 IU/kg/day; 28 days) revealed a strong attenuation of the spectrum of neuropathic manifestations. These behavioral improvements were linked to antioxidative effects in the spinal cord (L4-L6) and ipsilateral sciatic nerve, characterized by a significant reduction in MDA levels and restored SOD and GPx activity [244]. Vitamin D3 also attenuated neuroinflammation, decreasing numerous pro-inflammatory cytokines (TNF-α, IL-1β, IL-6) and increasing IL-10, while preserving axonal integrity and reducing microglial and macrophage activation [38].

In another CCI study, daily vitamin D (250–500 IU/kg/day, i.p.) or progesterone (4–6 mg/kg/day, i.p.) from postoperative day 1 to 21 showed limited efficacy alone; vitamin D did not alter nociception, and progesterone reduced allodynia but not thermal hyperalgesia. However, combined administration of vitamin D (500 IU/kg/day) and progesterone (6 mg/kg/day) significantly alleviated thermal hyperalgesia and mechanical allodynia. This synergistic effect likely reflects their complementary neurosteroid actions on neural repair pathways, although further studies are needed to confirm these mechanisms [245]. On the other hand, rats received oral supplementation with 60–400 IU/kg/day of vitamin D3, leading to a significant improvement in mechanical nociceptive thresholds in monoarthritic rodents, accompanied by strong reduction in mechanical hyperalgesia and cold allodynia [246].

Transitioning to more selective traumatic models, the spared nerve injury (SNI) paradigm has illuminated sensory fiber-specific mechanisms, causing stable mechanical allodynia mediated by sural A-low threshold mechanoreceptor sprouting without confounding thermal or motor deficits [247]. In rats, systemic vitamin D3 (1000 IU/kg/day, i.p.) or intrathecal vitamin D3 (10 μg/kg/day) initiated on postoperative day 1 and maintained for 14 consecutive days normalized the paw withdrawal threshold (PWT) to sham levels. This behavioral recovery occurred alongside inhibition of mitochondria-associated ferroptosis in the spinal dorsal horn (L4-L5), an iron-dependent lipid peroxidation cascade implicated in neuropathic disinhibition [38].

Lastly, in a vincristine-induced peripheral neuropathy (VIPN) rat model, vitamin D3 supplementation (500 IU/kg/day) promoted functional and structural recovery, such as improved electrophysiological outcomes and locomotor function, exceeding lower levels and highlighting its regenerative potential in toxic neuropathies [248].

### 5.2. Clinical Evidence

The clinical evidence regarding vitamin D supplementation has increasingly highlighted its significant role in modulating neurological, immunological, and musculoskeletal outcomes, particularly among patients experiencing chronic neuropathic pain, autoimmune disorders, or inflammatory conditions [250]. Vitamin D deficiency is highly prevalent among these populations, often correlating with greater symptom severity, impaired nerve conduction, heightened pain sensitivity, and suboptimal responses to conventional pharmacologic therapies [47,251].

Peripheral neuropathies of diverse etiologies, including CIPN, DPN, post-infectious neuropathies, and idiopathic neuropathies, have demonstrated responsiveness to vitamin D supplementation. Observational data indicate that patients with lower baseline vitamin D levels are more likely to develop neuropathic symptoms and report greater pain intensity [47,251,252]. Supplementation has been associated with significant reduction in many pain scores, reduced paresthesia, improved tactile and thermal sensation, and partial restoration of sensory thresholds (Table 2) [253,254,255,256,257,258,259,260,261,262].

Dosing regimens in clinical studies vary widely, reflecting differences in baseline deficiency, patient group, and therapeutic goals. Response to supplementation can be influenced by factors including age, body mass index, genetic polymorphisms in VDR or metabolic enzymes (e.g., CYP27B1 and CYP2R1), renal and hepatic function, and coexisting comorbidities [263,264]. Although vitamin D is generally safe, excessive dosing might result in hypercalcemia, nephrolithiasis, or renal dysfunction, highlighting the importance of clinician-guided administration and serial monitoring [265,266].

Vitamin D is increasingly recognized as an adjunctive therapy, complementing conventional pharmacologic treatments for neuropathic pain, including anticonvulsants, tricyclic antidepressants, gabapentinoids, and opioids [50,255]. Evidence shows that supplementation can enhance analgesic efficacy, reduce required dosages of standard medications, and mitigate dose-dependent adverse effects [40,250]. Integration of vitamin D into multimodal pain management frameworks, which include physical therapies, cognitive-behavioral interventions, lifestyle modifications, and dietary optimizations, has been associated with significant reductions in pain scores, improvements in physical and functional performance, and enhancements in psychosocial well-being [250,267].

Despite the growing body of robust clinical evidence, significant gaps persist in comprehensively delineating the full therapeutic scope of vitamin D. Longitudinal, multicenter trials with standardized dosing protocols, larger and more heterogeneous cohorts, and rigorous outcome measures are completely needed to clarify optimal treatment durations, dose-response relationships, and long-term safety [268]. Development of stratification algorithms incorporating genetic, biochemical, and clinical variables may enable predictive modeling of therapeutic responsiveness, thereby contributing to the continued refinement and advancement of precision medicine frameworks [269].

## 6. Conclusions

Neuropathic pain is a multifactorial condition sustained by complex molecular and cellular alterations affecting both the PNS and CNS. Peripheral nerve injury contributes to abnormal ion channel expression (particularly VGSCs and VGCCs), leading to ectopic discharges and increased neuronal excitability. At the spinal and supraspinal levels, persistent nociceptive input promotes central sensitization through enhanced glutamatergic transmission, NMDA receptor activation, attenuated inhibitory GABAergic signaling, and maladaptive synaptic plasticity. A key unifying mechanism in these processes is neuroinflammation, characterized by activation of microglia and astrocytes, increased release of pro-inflammatory cytokines, and activation of intracellular pathways. These mechanisms sustain neuronal hyperexcitability and contribute to the chronification of pain.

Within this pathophysiological context, vitamin D emerges as a biologically plausible modulator of neuropathic pain mechanisms. Via VDR activation, vitamin D regulates the transcription of genes involved in immune response, inflammatory signaling, oxidative stress control, and neuronal survival. Experimental findings suggest that vitamin D might attenuate neuroinflammation by downregulating NF-κB activity, reducing pro-inflammatory cytokine production, and limiting microglial activation. Moreover, its potential role in modulating Ca^2+^ homeostasis, supporting mitochondrial function, decreasing oxidative stress, and influencing neurotrophic factors further supports its relevance in the regulation of neuronal excitability and synaptic plasticity.

Clinically, the association between vitamin D deficiency and increased neuropathic pain severity across multiple patient populations reinforces the hypothesis of a meaningful interaction. Interventional studies show that supplementation might improve pain intensity and functional outcomes, particularly in individuals with documented deficiency. However, variability in study design, supplementation protocols, baseline vitamin D levels, and outcome measures restricts the ability to establish definitive conclusions regarding efficacy and causality.

Despite these promising mechanistic and clinical signals, several challenges remain that currently limit the translational application of vitamin D in neuropathic pain. These include the absence of standardized dosing protocols and variability in baseline vitamin D assessment, uncertainty regarding optimal serum target levels, and insufficient stratification of patient subgroups. Furthermore, most existing studies are limited by small sample sizes, short follow-up periods, and the absence of robust mechanistic endpoints, which collectively hinder the establishment of clear dose–response relationships and causal inferences.

From a future perspective, there is a need for large, well-designed randomized controlled trials to better determine the therapeutic value of vitamin D in neuropathic pain. Integrating mechanistic biomarkers with clinical outcomes may help identify responders and define patient-specific treatment strategies. Moreover, longitudinal studies exploring early intervention, combination therapies as part of multimodal pain management strategies, and the interaction between vitamin D status and neuroimmune phenotypes are essential in refining its clinical utility.

In conclusion, the evidence supports a clinically association between vitamin D and key molecular mediators of neuropathic pain, notably neuroinflammatory processes and central sensitization. While vitamin D should not be regarded as a primary analgesic intervention, optimizing its status might represent a rational adjunct within a multimodal, mechanism-based therapeutic strategy. Rigorous randomized controlled trials and translational studies are needed to elucidate causal pathways, identify patient subgroups most likely to benefit, and refine personalized treatment approaches.

## Figures and Tables

**Table 1:** Comprehensive synthesis of preclinical studies examining vitamin D_3_ as an intervention in rodent models of neuropathic pain, with emphasis on dosing protocols, delivery routes, treatment duration, and associated behavioral and molecular alterations. Abbreviations: CCI (chronic constriction injury); IU (international unit); i.p. (intraperitoneal); MDA (malondialdehyde); SOD (superoxide dismutase); GPx (glutathione peroxidase); TNF-α (tumor necrosis factor alpha); IL-1β (interleukin 1 beta); IL-6 (interleukin 6); IL-10 (interleukin 10); SNI (spared nerve injury); i.t. (intrathecal); VIPN (vincristine-induced peripheral neuropathy).

Model	Intervention	Duration	Effects and Mechanisms	References
CCI	Vitamin D3 (1000 IU/kg/day, i.p.)	3 weeks	Reduced thermal hyperalgesia and cold allodynia	[243]
	Vitamin D3, (oral 500 IU/kg/day)	4 weeks	Attenuated neuropathic behaviors Reduced MDA levels Restored SOD and GPx activities in spinal cord and ipsilateral sciatic nerve Reduced TNF-α, IL-1β, and IL-6 and increased IL-10 levels Preserved axonal integrity Reduced microglia and macrophage activation	[38,244]
	Vitamin D3 (250–500 IU/kg/day, i.p.) + Progesterone (4–6 mg/kg/day, i.p.)	3 weeks	Combination of vitamin D3 with progesterone significantly alleviated thermal hyperalgesia and mechanical allodynia	[245]
Monoarthritis	Vitamin D3 (oral 60–400 IU/kg/day)	6 weeks	Improved mechanical nociceptive thresholds Attenuated mechanical hyperalgesia and cold allodynia	[246]
SNI	Vitamin D3 (1000 IU/kg/day, i.p.) or (10 μg/kg/day, i.t.)	14 days	Normalized paw withdrawal threshold Inhibited mitochondria-associated ferroptosis in L4-L5 spinal dorsal horn	[38,247]
VIPN	Vitamin D3 (500 IU/kg/day, i.p.)	4 weeks	Improved locomotor function Promoted structural and functional recovery	[248]

**Table 2:** Overview of clinical investigations on vitamin D supplementation in neuropathic pain patients, detailing study populations, dosage regimens, intervention duration, and reported outcomes. Abbreviations: DPN (diabetic peripheral neuropathy); IU (international unit); 25(OH)D (25-hydroxyvitamin D or calcidiol); NSS (neuropathy symptom score); NDS (neuropathy disability score); NCS (nerve conduction study); i.m. (intramuscular); DN4 (Douleur neuropathique 4); SFMPQ (short-form McGill pain questionnaire); CRP (C-reactive protein); TNF-α (tumor necrosis factor alpha); VAS (visual analog scale); PDI (Pain Disability Index); BMI (body mass index); IL-6 (interleukin 6); IL-10 (interleukin 10); EMG (electromyography); BBT (Berg Balance Test); HbA1c (glycated hemoglobin); MNSI (Michigan neuropathy screening instrument); CIPN (chemotherapy-induced peripheral neuropathy); PO_4_^3−^ (phosphate ion; PTH (parathyroid hormone).

Population	Vitamin D Dose Regimen	Duration	Outcomes Observed	References
DPN (n = 112)	Oral vitamin D3 (50,000 IU, once weekly)	8 weeks	This prospective, placebo-controlled trial included 112 type 2 diabetic patients with diabetic peripheral neuropathy and vitamin D deficiency, assigned to vitamin D3 (n = 57) or placebo (n = 55) for 8 weeks. Vitamin D supplementation significantly increased serum 25(OH)D levels compared with placebo (32.8 ± 23.7 vs. 1.1 ± 3.6, *p* < 0.0001) and led to a greater reduction in NSS (−1.49 ± 1.37 vs. −0.20 ± 0.59, *p* < 0.001). No significant differences were observed in NDS or NCS between groups No adverse effects were reported	[253]
DPN (n = 143)	i.m. vitamin D3 (600,000 IU, single dose)	20 weeks	This study included 143 predominantly type 2 diabetic patients with neuropathic pain, of whom 58 (40.5%) had vitamin D deficiency. A single i.m. dose of 600,000 IU vitamin D was administered, with outcomes assessed over 20 weeks. Baseline scores included DN4 (3.0 ± 1.8), McGill pain score (21.2 ± 14.9), and SFMPQ (2.1 ± 0.9), with mean serum 25(OH)D of 31.7 ± 23.3 ng/mL. Post-treatment, 25(OH)D significantly increased to 46.2 ± 10.2 ng/mL (*p* < 0.0001), accompanied by significant reductions in DN4, total pain score, and SFMPQ (*p* < 0.0001) No adverse effects were reported	[254]
DPN (n = 68)	Oral vitamin D3 (5000 IU, once daily)	8 weeks	This controlled, open-label, randomized clinical trial (1:1 allocation) included 68 patients DPN. The experimental group received standard therapy plus oral vitamin D (5000 IU/day), while the control group received standard therapy alone over 8 weeks. After 8 weeks, the vitamin D group showed a greater reduction in pain compared with controls, including VAS (−3.34 ± 2.03 vs. −2.37 ± 2.2, *p* = 0.044) and burning pain (1.76 ± 7.16 vs. 6.18 ± 13.93, *p* = 0.046). Mood improvement was also higher in the intervention group (88.2% vs. 70.6%, *p* = 0.031). Serum 25(OH)D increased more significantly in the vitamin D group (40.02 ± 15.33 vs. 18.73 ± 6.88 ng/mL; *p* < 0.001), with a larger change from baseline (+24.14 ± 13.68 vs. +3.10 ± 4.20 ng/mL; *p* < 0.001). A negative correlation was observed between vitamin D levels and VAS scores (r = −0.403, *p* = 0.018) No adverse events were reported	[255]
DPN (n = 225)	Oral vitamin D3 (4000 IU, once daily)	12 weeks	This randomized controlled trial included 225 patients with DPN allocated to five groups: mindfulness + placebo, placebo, mindfulness alone, vitamin D, and mindfulness + vitamin D. The vitamin D regimen consisted of 4000 IU/day (28,000 IU/week) for 12 weeks, and mindfulness involved 12 sessions. Outcomes included PDI score, neuropathic pain severity, quality of life measures, and metabolic parameters At baseline, no significant differences were observed between groups. After 12 weeks, all active intervention groups improved except the placebo-only group. No significant differences were found between the vitamin D and mindfulness groups when analyzed separately. However, the combined vitamin D + mindfulness group showed significantly greater improvement than either intervention alone (*p* < 0.05). No significant effects were observed on fasting blood sugar, BMI, or energy intake (*p* > 0.05) No adverse effects were reported	[256]
DPN (n = 34)	Oral vitamin D3 (40,000 IU, once weekly)	24 weeks	This randomized study included 67 patients with type 2 diabetes mellitus and peripheral neuropathy (34 females), of whom 62 completed the trial. Participants were assigned to oral cholecalciferol 40,000 IU/week for 24 weeks. Outcomes included neuropathy severity (NSS, NDS, and VAS), cutaneous microcirculation, and pro-inflammatory markers (interleukins, CRP, and TNF-α). Vitamin D insufficiency was present in 78% of completers. After 24 weeks, the 40,000 IU/week group showed significant improvements in neuropathy severity (NSS, *p* = 0.001; NDS, *p* = 0.001; VAS, *p* = 0.001) and cutaneous microcirculation (*p* < 0.05), along with reduced IL-6 (2.5 vs. 0.6 pg/mL, *p* < 0.001) and increased IL-10 (2.5 vs. 4.5 pg/mL, *p* < 0.001) No adverse effects were reported	[257]
DPN (n = 57)	i.m. vitamin D3 (300,000 IU, single dose)	12 weeks	This study analyzed 57 of 258 screened patients with neuropathic pain, including 32 in the treatment group and 25 in the placebo group. Neuropathy was assessed using the DN4 questionnaire, EMG for polyneuropathy, and balance via the BBT test. The treatment group received a single i.m. injection of 300,000 IU vitamin D, while controls received saline, with outcomes reassessed after 12 weeks. Compared with placebo, the vitamin D group showed a significant reduction in total DN4 scores (*p* = 0.008) and significant improvement in BBT scores (*p* = 0.001). Subgroup analysis also demonstrated greater reductions in electric shock (*p* = 0.006) and burning sensation (*p* = 0.001) in the treatment group No adverse effects were reported	[258]
DPN (n = 60)	Oral vitamin D3 (50,000 IU, once weekly)	12 weeks	This quasi-experimental study included 60 patients with type 2 diabetes mellitus and painful diabetic neuropathy aged 30–65 years, of whom 58 completed the trial. Participants received oral vitamin D3 at a dose of 50,000 IU weekly for 12 weeks. Neuropathy was assessed using the MNSI questionnaire, alongside measurements of fasting plasma glucose, HbA1c, Ca^2+^, and serum vitamin D levels After 12 weeks, significant improvements were observed in HbA1c, serum vitamin D, and both MNSI questionnaire and physical examination scores (*p* < 0.001) No adverse effects were reported	[259]
DPN (n = 320)	i.m. vitamin D2 (5000 IU/kg, once every 2 weeks)	12 weeks	This multicenter, randomized, double-blind trial included adults with type 2 diabetes and diabetic peripheral neuropathy, with 320 patients analyzed: vitamin D2 group (n = 154) and placebo group (n = 166). Participants received intramuscular vitamin D2 (5000 IU/kg biweekly) or placebo for 3 months, alongside standard diabetic care At 3 months, the effective rate was higher in the vitamin D group compared with placebo (75% vs. 62%, χ^2^ = 59.86, *p* = 0.0001), and this difference persisted at 1 year (65% vs. 38%, χ^2^ = 23.28, *p* = 0.0001). Serum 25(OH)D levels increased significantly in the intervention group compared with controls at both 3 months (59.7 ± vs. 45.6 nmol/L, *p* = 0.001) and 1 year (55.5 vs. 44.7 nmol/L, *p* = 0.001). Limb nerve conduction velocity improved more in the vitamin D group at 1 year (57.6 vs. 44.7 m/s, *p* = 0.0001). No serious adverse effects were reported, with mild side effects occurring in 4% of treated patients Six of 154 patients (4%) had nausea, pruritus, ostealgia, metallic taste in the mouth	[260]
DPN (n = 150)	Oral vitamin D (60,000 IU, once weekly)	24 weeks	This prospective, randomized controlled trial included 150 patients with type 2 diabetes mellitus, allocated into three groups (n = 50 each): oral hypoglycemic agents (group 1), empagliflozin (group 2), and empagliflozin + vitamin D (group 3). Outcomes were assessed over 6 months using biochemical parameters and neuropathic pain scales (DN4, Neuropathic Pain Symptom Inventory, and Ipswich Touch Test) Baseline characteristics included a mean age of 50 ± 6 years, diabetes duration of 15 ± 4.5 years, and HbA1c > 10% across groups. Serum vitamin D levels increased significantly by 64.7% in group 3 (19 ± 5 to 54 ± 8 ng/mL; *p* < 0.05). A significant reduction in HbA1c (−17.4%) was observed only in group 3, while no significant changes occurred in groups 1 and 2. Neuropathic symptoms improved significantly only in the empagliflozin + vitamin D group No adverse events were reported	[261]
DPN (n = 80)	i.m. vitamin D (200,000 IU, once monthly)	3 months	This case-control study included 89 prediabetic individuals with peripheral neuropathy and a control group of prediabetics without peripheral neuropathy. Patients with peripheral neuropathy were evaluated using the DN4 and SFMPQ questionnaires, along with biochemical measurements including 25(OH)D, Ca^2+^, PO_4_^3−^, PTH, HbA1c, fasting glucose, postprandial glucose, and lipid profile. The PN group received intramuscular vitamin D3 (200,000 IU monthly) for 3 months, with reassessment thereafter Vitamin D levels were not associated with peripheral neuropathy severity at baseline. After supplementation, significant improvements were observed in glycemic parameters (*p* ≤ 0.001) and neuropathic outcomes, with DN4 and SFMPQ scores decreasing from 6.4 ± 1.6 and 28.3 ± 7.2 to 2.5 ± 0.9 and 17 ± 6.3, respectively (*p* ≤ 0.001) No adverse events were reported	[262]
CIPN (n = 80)	Oral vitamin D2 (50,000 IU, once weekly)	8 weeks	In this two-arm randomized trial, 80 vitamin D- deficient patients starting taxane-based chemotherapy will be assigned to either prescribed vitamin D supplementation or standard care (5000 IU, daily oral vitamin D3) Serum vitamin D levels will be measured at weeks 4, 8, 12, and 24, with dose adjustments as needed, and neuropathic pain will be assessed via self-report at baseline, week 12, and week 24	[NCT05259527]

## Data Availability

Not applicable. No new data were generated.

## Abbreviations

The following abbreviations are used in this manuscript:

1,25(OH)_2_D	1,25-dihydroxyvitamin D (calcitriol)
25(OH)D	25-hydroxyvitamin D (calcidiol)
Akt	Protein kinase B
AMPA	α-amino-3-hydroxy-5-methyl-4-isoxazolepropionic acid
ATP	Adenosine triphosphate
BAX	BCL2-associated X protein
BBB	Blood-brain barrier
BBT	Berg Balance Test
BCL2	B-cell lymphoma 2
BDNF	Brain-derived neurotrophic factor
BMI	Body mass index
CA1	Cornu Ammonis region 1
CA2	Cornu Ammonis region 2
Ca^2+^	Calcium ion
CA3	Cornu Ammonis region 3
CBP	CREB-binding protein
CCI	Chronic constriction injury
CD68	Cluster of differentiation 68
Cdc42	Cell division control protein 42 homolog
c-Fos	FBJ murine osteosarcoma viral oncogene homolog
CIPN	Chemotherapy-induced peripheral neuropathy
Cl^−^	Chloride ion
CNS	Central nervous system
COX-2	Cyclooxygenase-2
CREB	cAMP response element-binding protein
CRP	C-reactive protein
CSF	Cerebrospinal fluid
CX3CL1	C-X3-C motif chemokine ligand 1
CYP24A1	Cytochrome P450 Family 24 subfamily A member 1
CYP27B1	Cytochrome P450 family 27 subfamily B member 1
CYP2R1	Cytochrome P450 2R1
DAMP	Danger-associated molecular pattern
DN4	Douleur neuropathique 4
DNA	Deoxyribonucleic acid
DPN	Diabetic peripheral neuropathy
DRG	Dorsal root ganglion
EAAT1	Excitatory amino acid transporter 1
EAAT2	Excitatory amino acid transporter 2
Egr-1	Early growth response protein 1
EMG	Electromyography
ER	Endoplasmic reticulum
ERK	Extracellular signal-regulated kinase
FBS	Fasting blood sugar
FGF23	Fibroblast growth factor 23
GABA	Gamma-aminobutyric acid
GDNF	Glial cell line-derived neurotrophic factor
GPx	Glutathione peroxidase
H3K27ac	Histone H3 lysine 27 acetylation
H3K4me3	Histone H3 lysine 4 trimethylation
H3K9ac	Histone H3 lysine 9 acetylation
HbA1c	Glycated hemoglobin
HDAC	Histone deacetylase
i.m.	Intramuscular
i.p.	Intraperitoneal
i.t.	Intrathecal
Iba1	Ionized calcium-binding adapter molecule 1
IFN-γ	Interferon gamma
IL-10	Interleukin 10
IL-12	Interleukin 12
IL-17	Interleukin 17
IL-18	Interleukin 18
IL-1β	Interleukin 1 beta
IL-2	Interleukin 2
IL-23	Interleukin 23
IL-6	Interleukin 6
iNOS	Inducible nitric oxide synthase
IU	International unit
IκBα	Nuclear factor of kappa light polypeptide gene enhancer in B-cells inhibitor alpha
K^+^	Potassium ion
KCC2	Potassium-chloride cotransporter 2
KCNB1	Potassium voltage-gated channel subfamily B member 1
KCNQ2	Potassium voltage-gated channel subfamily Q member 2
LEF	Lymphoid enhancer-binding factor
lncRNA	Long non-coding RNA
LRP2	Low-density lipoprotein receptor-related protein 2
M1	Classically activated macrophage/microglial phenotype
M2	Alternatively activated macrophage/microglial phenotype
MAP2	Microtubule-associated protein 2
MAPK	Mitogen-activated protein kinase
MDA	Malondialdehyde
MHC-II	Major histocompatibility complex class II
MNSI	Michigan neuropathy screening instrument
miRNA	MicroRNA
MMP-9	Matrix metalloproteinase 9
mtDNA	Mitochondrial DNA
Na^+^	Sodium ion
NADPH	Nicotinamide adenine dinucleotide phosphate (reduced form)
NaPi-IIa	Sodium-phosphate cotransporter type IIa
NaPi-IIc	Sodium-phosphate cotransporter type IIc
Nav1.7	Voltage-gated sodium channel alpha subunit 1.7
Nav1.8	Voltage-gated sodium channel alpha subunit 1.8
Nav1.9	Voltage-gated sodium channel alpha subunit 1.9
NCoR	Nuclear receptor corepressor
ncRNA	Non-coding RNA
NCS	Nerve conduction study
NDS	Neuropathy disability score
NF-κB	Nuclear factor kappa-light-chain-enhancer of activated B cells
NGF	Nerve growth factor
NHANES	National health and nutrition examination survey
NLGN	Neuroligin
NLRP3	NOD-like receptor family pyrin domain-containing 3
NMDA	N-methyl-D-aspartate
NO	Nitric oxide
NOX2	NADPH oxidase 2
Nrf2	Nuclear factor erythroid 2-related factor 2
NRXN	Neurexin
NSS	Neuropathy symptom score
NT-3	Neurotrophin 3
OPC	Oligodendrocyte precursor cell
OPG	Osteoprotegerin
p21	Cyclin-dependent kinase inhibitor 1
p27	Cyclin-dependent kinase inhibitor 1B
P2X4	Purinergic receptor P2X ligand-gated ion channel 4
PDI	Pain Disability Index
PDIA3	Protein disulfide isomerase family A member 3
PGC-1α	Peroxisome proliferator-activated receptor gamma coactivator 1 alpha
PI3K	Phosphoinositide 3-kinase
PKC	Protein kinase C
PMCA1b	Plasma membrane Ca^2+^ ATPase 1b
PNS	Peripheral nervous system
PO_4_^3−^	Phosphate ion
PSD-95	Postsynaptic density protein 95
PTH	Parathyroid hormone
PWT	Paw withdrawal threshold
Rac-1	Ras-related C3 botulinum toxin substrate 1
RANKL	Receptor activator of nuclear factor κB ligand
RhoA	Ras homolog family member A
RNA	Ribonucleic acid
ROS	Reactive oxygen species
RXR	Retinoid X receptor
SCN10A	Sodium voltage-gated channel alpha subunit 10
SCN11A	Sodium voltage-gated channel alpha subunit 11
SFMPQ	Short-form McGill pain questionnaire
SMRT	Silencing mediator for retinoid and thyroid hormone receptor
SNI	Spared nerve injury
SOD	Superoxide dismutase
SRC-1	Steroid receptor coactivator 1
SRC-2	Steroid receptor coactivator 2
SRC-3	Steroid receptor coactivator 3
TCF	T-cell factor
TGF-β	Transforming growth factor beta
Th1	T helper type 1 cell
Th17	T helper type 17 cell
TLR	Toll-like receptor
TNF-α	Tumor necrosis factor alpha
Treg	Regulatory T cell
Trk	Tropomyosin receptor kinase
TRP	Transient receptor potential channel
TRPA1	Transient receptor potential ankyrin 1
TRPV1	Transient receptor potential vanilloid 1
TRPV5	Transient receptor potential vanilloid 5
TRPV6	Transient receptor potential vanilloid 6
UVB	Ultraviolet B radiation
VAS	Visual analog scale
VDBP	Vitamin D binding protein
VDR	Vitamin D receptor
VDRE	Vitamin D response element
VEGF	Vascular endothelial growth factor
VIPN	Vincristine-induced peripheral neuropathy
VGCC	Voltage-gated calcium channel
VGSC	Voltage-gated sodium channel
Wnt	Wingless/Integrated signaling pathway
ZO-1	Zonula occludens 1
